# The Australian Feeding Infants and Toddlers Study (OzFITS) 2021: Study Design, Methods and Sample Description

**DOI:** 10.3390/nu13124524

**Published:** 2021-12-17

**Authors:** Najma A. Moumin, Rebecca K. Golley, Chelsea E. Mauch, Maria Makrides, Tim J. Green, Merryn J. Netting

**Affiliations:** 1 Discipline of Pediatrics, Faculty of Health and Medical Sciences, University of Adelaide, Adelaide, SA 5000, Australia; najma.moumin@sahmri.com (N.A.M.); maria.makrides@sahmri.com (M.M.); tim.green@sahmri.com (T.J.G.); 2 Women and Kids Theme, South Australian Health and Medical Research Institute, Adelaide, SA 5000, Australia; 3 Caring Futures Institute, College of Nursing and Health Sciences, Flinders University, Adelaide, SA 5000, Australia; rebecca.golley@flinders.edu.au (R.K.G.); chelsea.mauch@flinders.edu.au (C.E.M.); 4 Nutrition Department, Women’s and Children’s Health Network, Adelaide, SA 5006, Australia

**Keywords:** Australian feeding infants and toddlers study, complementary feeding, nutrient intakes, feeding practices

## Abstract

(1) Background: Caregiver feeding practices during the first two years of a child’s life influence nutrition, growth, and development, as well as long term taste preferences and dietary patterns. Suboptimal feeding practices lead to poorer health outcomes, such as obesity, that persist into adulthood. Although the importance of early life nutrition is well-established, there are no Australia-wide surveys of dietary intakes of children under two years of age. The 2021 Australian Feeding Infants and Toddlers Study (OzFITS) aims to fill this gap. This paper describes the methods and study sample of OzFITS 2021. (2) Methods: OzFITS 2021 is a cross-sectional study of children aged 0 to 23.9 months of age and their caregiver across Australia. Data were collected between April 2020 and April 2021. A telephone-based survey was completed with a caregiver to obtain information on child and caregiver characteristics and feeding practices. For exclusively breastfed infants, the number of breastfeeds in a 24 h period was reported. Dietary intakes for mixed fed children were estimated using a one-day food record, with 30% of caregivers completing a second food record on a non-consecutive day. (3) Results: We enrolled 1140 caregiver and child dyads. Of those eligible to complete a food record, 853 (87%) completed the food record. Compared to the Australian population, caregivers were more likely to be university-educated (>75%), married or in a de facto relationship (94%), and have a household income >$100,000/y (60%). (4) Conclusions: OzFITS 2021 is the first national study to examine food and nutrient intake in Australian children aged under 2 years. The study will provide information on breastfeeding rates and duration, use of breast milk substitutes, and timing of solid food introduction. Dietary intake data will allow the comparison of core food groups and discretionary food intake to Australian guidelines and estimate the prevalence of inadequate intake of key nutrients, like iron. Healthcare practitioners and policymakers can use the study findings as a source of evidence to inform the next iteration of infant feeding guidelines.

## 1. Introduction

During the first two years of life, caregiver feeding practices influence a child’s growth and development along with their long-term taste preferences, dietary patterns, and appetite regulation [1,2,3,4]. Suboptimal feeding practices and nutrition in early childhood contribute to malnutrition and poor child health outcomes that may persist into adulthood [5,6]. During this period, nutrient or energy deficiencies may lead to increased disease risk, stunting, and impaired cognitive development [5,7]. Conversely, excess weight gain during the first two years is associated with overweight and obesity and chronic disease later in life [8].

Health authorities in many countries give guidance on infant and toddler feeding and nutrition [9,10,11,12,13,14]. Most emphasize exclusive breastfeeding for the first six months of life, followed by the introduction of complementary foods at around six months, emphasizing iron-rich foods and continued breastfeeding to two years and beyond [2,9,10,11,12,13,14,15]. Further, caregivers are advised to offer infants a range of nutritious foods with increasing complexity of textures so that children can eat mainly family foods by one year of age [11,12]. More recently, deliberate introduction to and regular intake of common food allergens, specifically egg and peanut has been encouraged prior to one year of age as a primary allergy prevention strategy [16]. Despite the recognized importance of early childhood feeding and nutrition, limited data exist on caregiver feeding practices and dietary intakes of Australian children under two years of age.

Over the last 30 years, three national nutrition surveys have been conducted in Australia, but none have included children less than 24 months of age [17,18,19]. Although the 2010 Australian National Infant Feeding Survey captured breastfeeding rates and early caregiver feeding practices, including the age of introduction of solid foods, it did not capture the types of foods and drinks consumed by young Australians [20]. The few studies that have reported dietary intake in this age group suggest that infant feeding practices do not meet current guidelines; however, these studies were only in select cities and are over a decade old [21,22,23,24].

Given the importance of early life feeding practices on short- and long-term health outcomes, it is surprising that there is no national data on the contemporary feeding practices of young children. To address this gap, in this study, we aim to: (1) describe breastfeeding initiation rates, duration, and use of breast milk substitutes; (2) determine the timing of introduction of solid foods including common food allergens; (3) estimate usual energy and nutrient intake distribution and prevalence of inadequate nutrient intakes; and (4) compare dietary intake of core food groups and discretionary foods to Australian Guidelines for Healthy Eating [12]. Here, we report the methods used for the 2021 Australian Feeding Infants and Toddlers Study (OzFITS) in the papers that follow. Specifically, we describe the study design, recruitment method, instrument development and testing, sampling frame, data collection, and key characteristics of the participants.

## 2. Materials and Methods

### 2.1. Study Design

The 2021 OzFITS study was a cross-sectional survey of a convenience sample of Australian infants and toddlers 0–24 months of age. The study was conducted between April 2020 and April 2021 and consisted of a telephone-based socio-demographic and child-feeding questionnaire and a food record completed by the child’s caregiver. The Women and Children’s Health Network Human Research Ethics Committee (HREC/19/WCHN/44) approved the study, and all caregivers gave informed verbal consent. 

### 2.2. Pilot Testing

Prior to commencing the study, data collection instruments were developed, tested, and revised. The socio-demographic and child-feeding questionnaire, portion estimation guide, and food record (Section 2.4.3.1) were pilot tested in a group (*n* = 48) of caregivers with children aged under 2 years in Adelaide. Participant feedback was used to revise the survey questions for clarity, add additional questions, develop processes to improve the food record’s user experience and streamline participant flow. A data quality audit was performed of all food records and assessed each child’s energy intake against their Estimated Energy Requirements (EER) [25].

The feasibility of a random selection recruitment method was also piloted. Postcodes were stratified according to the socioeconomic index for areas index and ranked into tertiles of relative advantage and disadvantage [26]. Postcodes were then randomly selected based on probability proportional to population size. Flyers were sent to all households within randomly selected postcodes in South Australia, inviting them to participate in the study if they had a child aged less than two years. Of 17,200 brochures sent out, only 16 participants responded. Because of the desired sample size and study timeline, this recruitment method was deemed infeasible. Therefore, the recruitment method described in Section 2.3 was adopted.

### 2.3. Sampling Frame

Caregivers were recruited through targeted online advertising using a trial recruitment company, Trialfacts [27]. Caregivers were pre-screened for eligibility. To be included they needed to (1) have a child between 0–24 months; (2) be knowledgeable about the child’s diet and able to answer questions about the child from birth; (3) have basic English fluency and able to provide informed consent. Infants or toddlers with significant health issues that affected feeding were excluded.

The sampling frame included all Australian states and territories, with the target sample within each state and territory proportional to its population size. A major aim of our study was to estimate the prevalence of inadequacy of select nutrients, with emphasis on iron, an essential limiting nutrient in this age group. Due to a lack of representative Australian data, the expected proportion of the population with inadequate intakes were based on FITS 2016 data [28]. Assuming an overall population size of 150,000 infants 6–12 months and 300,000 toddlers 12–24 months [29], we estimated a minimum sample size of 227 and 100 caregiver-child pairs, respectively, to determine the true prevalence of inadequacy for iron to within ±5% and 95% confidence. For all other nutrients, the required sample size ranged from 1 for vitamin C to 126 for calcium. Due to uncertainty around prevalence estimates and to maintain the Type 1 error rate at 5% with multiple estimates, we enrolled 250 children per six-month age band: 0–5.9 months; 6–11.9 months; 12–17.9 months; and 18–23.9 months.

### 2.4. Data Collection

#### 2.4.1. Socio-Demographic and Child-Feeding Questionnaire

The socio-demographic and child-feeding questionnaire questions were adapted from the 2016 United States Feeding Infants and Toddlers Study (FITS) and the 2010 Australian National Infant Feeding Survey [20,30]. As 1 in 10 Australian infants aged 12 months has a confirmed food allergy [31], a module on the timing of allergen introduction and prevalence of food allergy was included. Eligible caregivers were asked to schedule a time to be contacted by study staff. Up to five phone call attempts were made to contact caregivers of eligible children.

Verbal informed consent was obtained from the caregiver before commencing the phone survey. The survey consisted of six modules: (1) caregiver socio-demographics; (2) childbirth details and anthropometric measures; (3) breastfeeding history and use of breastmilk substitutes; (4) timing of introduction of complementary foods and common allergens; (5) use of commercial baby foods and mode of feeding; and (6) dietary supplement use. Once the survey was complete, the caregiver received a $20 supermarket gift card. Following the survey, eligible caregivers were asked to complete a food record and participate in a follow up phone interview to collect data of child food intake. Caregivers of children who were exclusively breastfed did not complete this module. Instead, a detailed breastfeeding history including the average number of breastfeeds in a 24-h period were collected. All survey data were collected and managed using REDCap™ (Research Electronic Data Capture), a secure, web-based software platform that supports data capture, audit, and export for research studies [32,33]. Study staff received a two-day training seminar on the survey instrument and the REDCap™ data entry system. 

#### 2.4.2. Child Anthropometry

Child length (cm) and weight (kg) measurements taken by a health professional in the previous 30 days were used. If these were not available, caregivers were asked to measure their child’s length and weight at home. Instructions adapted from the WHO Training Course on Child Growth Assessment were sent to caregivers [34]. 

#### 2.4.3. Dietary Assessment Method

Food and beverage intake was assessed using a 1-day food record, with repeats in a random subset (30% of the sample), followed by a phone call to review the food record for accuracy and completeness. A 24-h dietary recall method was not used because this method relies on participant recall of intake from the previous day and is prone to over-estimation of true intake, which, for this age group would significantly impact results [35,36]. Further, a large proportion of children in this age group are often cared for and fed by other people (i.e., childcare workers and grandparents), and the caregiver would not necessarily be able to recall what the child had eaten the previous day. The food record on the other hand allowed multiple caregivers to record food intake. A validated portion estimation guide, training prior to record collection, and real time data capture helped minimize errors with portion estimation [37]. 

##### 2.4.3.1. Food Record

Caregivers were randomly assigned to a day of the week to complete a food record for their child. A ~30% subsample of the study population was randomly selected to complete a second day’s intake on a non-consecutive day, to allow estimation of usual intake distribution. The randomization schedule was developed by a statistician to ensure a balance of weekdays and weekend days. Caregivers were asked to record everything the child consumed from midnight the previous day to midnight the following day. A study package including a food record booklet, and a portion estimation guide [17,30] were mailed to the caregivers to help them record the child’s intake. Additional food record booklets were included if the child was under someone else’s care.

Once the study package was received, staff completed a preparatory phone call with the caregivers. Caregivers were instructed on how to record portion sizes of foods offered and amounts of food left uneaten using standard metric cup and spoon measures available in the home, or alternatively using gram measurements if household kitchen scales were available. If neither of these options were feasible, staff explained the portion estimation guide in detail. An example entry day was also reviewed with caregivers to ensure understanding of the level of detail required in describing food items including how to record household recipes. A copy of the instructions was also included with the secondary forms along with study staff contact information should questions arise. Once completed, caregivers were asked to take photos of the booklet(s) and scan or email them back to study staff. Reminder text messages, phone calls, and emails were sent up to a maximum of five times before participants were classified as lost to follow up.

##### 2.4.3.2. Follow-Up Call

After the food record(s) were returned, study staff scheduled a phone appointment with caregivers. Interview techniques outlined in the four pass 24-h recall method were used to review the record [38]. The food record served as pass one, where all foods and drinks listed were reviewed and confirmed. Pass two entailed obtaining a detailed description of each listed item i.e., brand name, variety, preparation methods, and/or additions at the time of consumption. The third pass involved confirming portion sizes and the number of serves including amounts leftover or spilled. If quantities appeared implausible or incomplete, the portion estimation guide was used to help clarify quantities. Finally, the fourth pass involved reviewing the record, probing for forgotten foods and drinks and determining how usual the intake for the day was (e.g., illness, party etc.). If the caregiver reported food/drink intake to be more or less than usual, interviewers recorded the reasons provided. Caregivers received an additional $20 supermarket gift card upon completion of the food record and follow-up interview.

##### 2.4.3.3. Handling of Food Intake Data

All food intake data was entered directly into FoodWorks™ Professional version 10 [39]. This program used the 2011-13 Australian Food, Supplement and Nutrient Database (AUSNUT) developed during the National Nutrition and Physical Activity Survey [40]. As most commercial infant and toddler foods were not included in the AUSNUT database, we developed our own infant and toddler foods database [41]. The database was periodically updated as new products became available. Nutrient information and ingredient lists for these foods were obtained directly from product packaging or vendor/manufacturer websites. Foods and household recipes with less than four ingredients were entered as individual food items. Household recipes with more than four ingredients were added as a recipe; the amount consumed by the child was then calculated as a proportion of the total recipe. For commercial products, the amount of a micronutrient was only on the nutrition information panel if a nutrient claim was made; therefore, a recipe approach based on product ingredient lists was used to estimate the micronutrient content of most foods [42]. Briefly, ingredients were entered as cooked food items in FoodWorks™ based on their proportion within the ingredient list (product recipe). Where a single percentage was reported for multiple ingredients, different quantities were imputed for individual ingredients until a nutrient profile that closely matched the nutrition information panel was achieved. All product recipes imputed in this way were within 10% of the manufacturer reported energy, total fat, carbohydrate, protein, and total sugars. For fortified products, nutrient values generated by FoodWorks™ were replaced with manufacturer reported values. 

All commercial foods and formula added to FoodWorks as new items (*n* = 301) were assigned a unique 8-digit food code based on AUSNUT 2011-13 major and sub-major food group classifications [40]. If home prepared recipes closely matched existing recipes in the AUSNUT 2011-13 recipe database, the same code was applied. Otherwise, unique codes were assigned based on the major food group or the ingredient accounting for the largest proportion within the recipe in the case of mixed food group recipes. Additional steps were also taken when coding recipes and commercial food items to categorize them as either discretionary or non-discretionary according to the Australian Bureau of Statistics discretionary food flag list [43]. The same principles outlined in the Australian Health Survey were used to systematically assess recipes and products [43].

Breastmilk intakes were estimated using validated assumptions used in previous studies developed in the United Kingdom (UK) and adapted in previous Australian studies [22,44,45]. The number of minutes the child actively suckled from the breast was recorded for each feed. A time of 10 min was recorded for feeds exceeding 10 min or when feeds occurring within 30 min of one another totaled over 10 min. Breastfeeds less than two minutes were excluded. The mass flow rate of breastmilk was estimated to be 10 g/min and energy intake was assumed to be 2.77 kJ/g [25,44]. 

Infant formula and toddler milk intakes were calculated from information in the food record, specifically, the number of scoops added to a volume of water, the prepared volume offered, and the prepared volume remaining in the bottle after a feeding. The gram weight of infant formula or toddler milk consumed was then determined by the following equation: formula (g) = scoop weight formula (g)/prepared volume (mL) × consumed volume (mL). 

Food intake data was entered by one of two trained interviewers. The training included a comprehensive review of OzFITS 2021 methods, study protocols, and data entry rules for the follow-up interviews. Investigators completed a data quality audit of 10% of food records. Selected food records and their corresponding entries in FoodWorks™ were compared for consistency. Any obvious mistakes such as a missed food was corrected during the audit. Any unclear quantities were investigated further by reviewing case notes and discussing the entry with the respective interviewer. For outliers with extremely low or very high energy intakes, food records were reviewed by an investigator (MN) to ascertain the plausibility of reported intakes.

### 2.5. Data Analysis

Descriptive statistics—specifically, frequencies, means and standard deviations were used to report sample characteristics. As loss to follow up and withdrawal was low (10.9%), only cases with valid responses were included in the analysis. All statistical analysis was completed using SPSS version 28.0 [46]. Data analysis for the remainder of the papers in this supplement will be presented in the papers that follow.

## 3. Results

### 3.1. Sample Size and Response Rate

**Figure 1:**
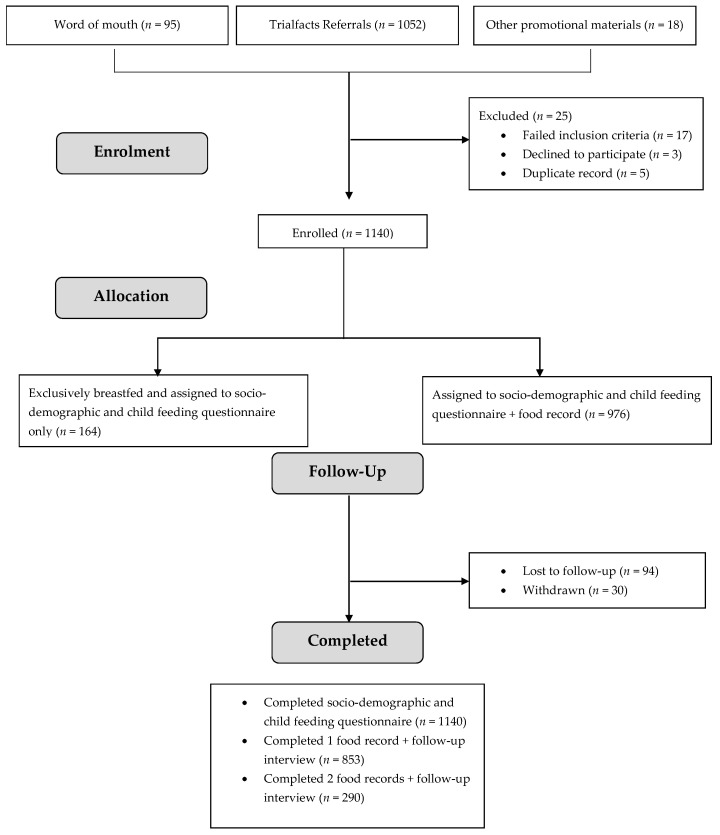
Participant flow and follow-up for OzFITS 2021.

Study recruitment occurred between April 2020 and April 2021. In total, 1140 caregivers were enrolled. Over 90% of caregivers were recruited through the online recruitment agency (Figure 1). After screening for eligibility, 17 caregivers were ineligible, three were eligible but declined to participate, and five had already participated. Completion rates for each phase of the study by age band are described in Table 1. Of the 976 caregivers asked to complete a food record, 87% completed at least a single day. A total of 345 (35%) of caregivers were asked to complete a second day’s food record, and 290 (84%) did so. 

### 3.2. Sample Characteristics

The key characteristics of the sample are presented in Table 2. The average age of caregivers was 34.2 ± 4.5 years and nearly all (98%) were the child’s biological mother. Over 75% of respondents had an undergraduate degree or above, 61% reported an annual household income greater than $100,000, and over 90% were married or living with a partner in a de facto relationship. Around 70% of the respondents were born in Australia and the remainder were born overseas. Of those born overseas, the majority were from the UK, Vietnam, New Zealand, Malaysia, and India. 

## 4. Discussion

OzFITS 2021 is the first comprehensive assessment of dietary intakes of a national sample of Australian infants and toddlers. Based on the 2016 US FITS study, OzFITS 2021 benefits from the methodological rigor developed over the past three iterations of the survey. Data from OzFITS 2021 will fill an important evidence gap by providing contemporary data on the following: breastfeeding duration and use of breast milk substitutes; timing and introduction of solid foods; consumption of core food groups and discretionary foods; and nutrient intakes. OzFITS 2021 will also allow the identification of new and emerging trends in infant feeding practices in Australia. 

There are study design and methods limitations. Due to feasibility, the survey used a convenience sampling instead of a random sampling method. The impact of this sampling method is selection bias, which in this study was towards caregivers from high SES groups. Compared to the 2016 Australian population, the OzFITS 2021 sample was more likely to be university educated (76% vs. 22%) and report higher annual household incomes (>$100,000 vs. $74,776) [47]. Since maternal education and household income are known to be positively associated with nutrition status, our results may not be representative of the wider population [5]. The National Nutrition and Physical Activity Survey required a complex sampling frame identifying eligible households and took considerable financial and human resources to obtain a representative sample of Australians. 

Another potential limitation is that the food record removed the element of surprise; however, this method improves portion size estimation and reduces recall bias. Unlike studies of intake in adults where intake is often underestimated, this does not seem to be an issue when estimating intake in infants and toddlers [28]. However, social desirability bias in our study may have influenced food choices and led to under- or overestimation of discretionary or healthful foods, respectively [48]. Finally, data collection occurred during the SARS-CoV-2 pandemic in which many Australians were locked down and may have influenced feeding practices. However, restrictions were short lived in most Australian cities, excluding Melbourne, and most caregivers did not report any impact of the pandemic on feeding practices for their children. 

In conclusion, the OzFITS 2021 used robust methods to report caregiver feeding practices and dietary intakes of Australian children 0–23.9 months of age. For the first time, nutrient intake, and prevalence of inadequate intake for a range of nutrients is available for a national sample of infants and toddlers. Results from the OzFITS study can be used to inform future iterations of national infant feeding guidelines and interventions to optimize early life feeding. Ultimately, OzFITS provides the necessary tools and framework needed to implement a nationally representative nutrition survey within this age group.

## Figures and Tables

**Table 1:** Completed questionnaire, food record, and follow up interview by age band for infants and toddlers enrolled in OzFITS 2021 (*n* = 1140).

Age Group, Months	Child-Feeding Questionnaire	Eligible for Food Record, *n* (%)	Assigned to Complete a Second Food Record, *n* (%)	Completed Food Record	
				One Day Food Record, *n* (%)	Two-Day Food Record, *n* (%)
0–5.9	290 (25.4)	126 (43.4)	47 (37.3)	114 (90.5)	46 (97.9)
6–11.9	308 (27.0)	308 (100)	110 (35.7)	279 (90.6)	98 (89.1)
12–17.9	289 (25.4)	289(100)	99 (34.3)	245 (84.8)	75 (75.8)
18–23.9	253 (22.2)	253 (100)	89 (35.2)	215 (85.0)	71 (80.0)
Total	1140(100)	976 (85.6)	345 (35.3)	853 (87.4)	290 (84.1)

**Table 2:** Demographic characteristics of 2021 OzFITS sample (*n* = 1140).

Demographic Characteristics	2021 OzFITS Sample
Household size, *n* (%)	
2	34 (3.0)
3	616 (54.0)
4	337 (29.6)
>4	151 (13.2)
Australian State, *n* (*%)*	
Australian Capital Territory	30 (2.6)
New South Wales	333 (29.2)
Northern Territory	9 (0.8)
Queensland	205 (18.0)
South Australia	119 (10.4)
Tasmania	30 (2.6)
Victoria	312 (27.4)
Western Australia	102 (8.9)
Annual Household Income (AUD), *n* (%)	
<$40,000	50 (4.4)
$40,001–$70,000	113 (9.9)
$70,001–$105,000	267 (23.4)
$105,001–$205,000	537 (47.1)
>$205,000	158 (13.9)
Prefer not to disclose	15 (1.3)
Caregiver age, y (mean ± SD)	34.2 ± 4.5
Relationship to the child *n* (%)	
Biological Mother	1112 (97.5)
Other	28 (2.5)
Parity *n* (%)	
Only child	678 (59.5)
2 children	355 (31.1)
>2 children	107 (9.4)
Born in Australia *n* (%)	804 (70.5)
Aboriginal and Torres Strait Islander *n* (%)	10 (0.9)
Marital Status *n* (%)	
Married/de facto	1073 (94.1)
Other	67 (5.8)
Educational Attainment, *n* (%)	
Year 10 or 11	8 (0.7)
Secondary school	53 (4.6)
Certificate or Diploma	218 (19.1)
Bachelor degree or above	861 (75.5)
Employment Status, *n* (%)	
Parental leave	50 (4.4)
Caring duties	159 (13.9)
Employed full time or part-time	903 (79.2)
Student	20 (1.8)
Other	8 (0.7)

## Data Availability

The data presented in this study are available on request from the corresponding author.
